# Conversion of Waste Polyethylene Terephthalate (PET) Polymer into Activated Carbon and Its Feasibility to Produce Green Fuel

**DOI:** 10.3390/polym13223952

**Published:** 2021-11-16

**Authors:** Firdous Ahmad Ahangar, Umer Rashid, Junaid Ahmad, Toshiki Tsubota, Ali Alsalme

**Affiliations:** 1 Institute of Nanoscience and Nanotechnology (ION2), University Putra Malaysia (UPM), Serdang 43400, Selangor, Malaysia; firdous45@gmail.com; 2 Faculty of Science and Technology, Free University Bozen-Bolzano, Piazza Universita 5, 39100 Bolzano, Italy; juni.utp@gmail.com; 3 Department of Materials Science, Graduate School of Engineering, Kyushu Institute of Technology, 1-1 Sensuicho, Tobata-ku, Kitakyushu, Fukuoka 804-8550, Japan; tsubota@che.kyutech.ac.jp; 4 Chemistry Department, College of Science, King Saud University, Riyadh 1145, Saudi Arabia; aalsalme@ksu.edu.sa

**Keywords:** activated carbon, non-biodegradable, waste PET bottles, characterization, waste feedstocks

## Abstract

In this study, a novel idea was proposed to convert the polyethylene terephthalate (PET) waste drinking-water bottles into activated carbon (AC) to use for waste cooking oil (WCO) and palm fatty acid distillate (PFAD) feasibility to convert into esters. The acidic and basic char were prepared by using the waste PET bottles. The physiochemical properties were determined by employing various analytical techniques, such as field emission scanning electron microscopy (FESEM), thermogravimetric analysis (TGA), Fourier transform infrared (FTIR), Brunauer–Emmett–Teller (BET) and temperature-programmed desorption – ammonia/carbon dioxide (TPD-NH_3_/CO_2_). The prepared PET H_3_PO_4_ and PET KOH showed the higher surface area, thus illustrating that the surface of both materials has enough space for impregnation of foreign precursors. The TPD-NH_3_ and TPD-CO_2_ results depicted that PET H_3_PO_4_ is found to have higher acidity, i.e., 18.17 mmolg^−1^, due to the attachment of phosponyl groups to it during pretreatment, whereas, in the case of PET KOH, the basicity increases to 13.49 mmolg^−1^. The conversion results show that prepared materials can be used as a support for an acidic and basic catalyst for the conversion of WCO and PFAD into green fuel.

## 1. Introduction

The activities carried out by humans are having a huge impact on the environment. There are always dire consequences of an activity on the natural resources and environment, as we tend to interfere in the natural mechanism of recycling. Polymers have been increasingly popular in recent years and have been used widely in almost every field of life. Around 300 million tons of plastic waste is generated globally each year. The number of benefits obtained from the usage of polymers is phenomenal, and that is why they replaced glass in 1970s, but this does not negate the fact that they cause pollution to our environment [1]. Over the passage of time, they have become an important material of our society because benefits associated with them, such as ease of adaptability in design, thermostability, transparency, durability as compared to glass and low cost. The consumption of PET has shown a fast rate of growth, due to the increase in growth of the plastic bottle market [2]. Plastic solid wastes are accumulating day by day in our surroundings, and this is posing a huge problem in their disposal without contaminating the environment. The amount of PET that is being recycled in a number of manners in the past is less, but its potential to be converted into activated carbon has been explored recently [3,4]. PET falls into the category of non-biodegradable plastic waste and is being dumped in the landfills and oceans, which poses a great danger to ecology, wild life and human health [5]. Sardon and Dove [6] anticipated that, by 2050, the mass of plastic waste will be more than that of fish, because plastic trash continues to rise, along with rapid disposal and weak recycling methods. All of the data suggest that plastic pollution has already become a severe concern, with far greater consequences than previously assumed.

Malaysians are the biggest consumers of plastics in Asia and contribute the maximum in terms of dumping of PET waste in oceans. The amount of carbon emissions associated with plastic from various stages of production to burning amounted to 860 million tons in 2019, which is more than the annual emissions of Thailand, Vietnam and the Philippines combined [7]. Carbon materials or their precursors are a low-cost feedstock for preparation of carbon-based catalyst, thus improving its potential for use in biodiesel manufacturing processes [8]. PET char has been prepared for a long time and used for various purposes other than that of Biodiesel production. It has been used as a sieve for the cleaning of a gas mixtures containing O_2_, N_2_ and CO_2_; CH_4_ and char can also use a tar filtering medium [9]. The activated carbon (AC) prepared showed good capacity for adsorption, and certain selectivity for O_2_/N_2_ and CO_2_/CH_4_ was prepared from granulated PET and Cork Oak with pore mouth-narrowing, using CVD from Benzene [10]. PET has been mixed with other synthetic polymers, such as Polyacrylonitrile (PAN), to obtain char with more yield and better textural properties. The production of highly porous AC utilizing a synthetic polymeric mixture with PET and PAN as a precursor for the removal of pesticides, such as diuron and 4-chloro-2-methylphenoxyacetic acid (MCPA), from the aqueous phase is an intriguing characteristic of this work. There are two advantages of PET char: one is the cost, and the second is the efficiency, which is very high [11]. PET char has been used for the removal of phenolic compounds from industrial-waste waters, which have proved to be a threat to the environment, as the effluents are directly drained into the rivers. The adsorption capacity is better for the char pretreated with KOH(s) rather than NaOH(s). Post modification with urea does not affect the adsorption capacity much, as compared to cloth and cork char [10]. PET char has been used for the removal of arsenic in wastewater. Among the many types of plastic chars tested, PET and PVC mixed char removed the most arsenic from the stock solution, ranging from 71.6 to 99.4 percent. For 99.4 percent Ar adsorption, the optimal equilibrium time of 20 min with acidic medium (pH 6.0) at 1.5 g was the most preferred dose [12]. The char produced by loading PET with 5% of Fe_2_O_3_ and MgO has been found to be useful as a flame retardant, and this happens due to synergistic action of them [13]. PET char was employed to make improved epoxy resins, and the tensile strength, surface hardness and electrical characteristics of epoxy composite materials were determined [14].

Biodiesel (ester) is a mixture of fatty acid ester, which is produced from the oil/fats that come from any source. Ester has similar combustion properties to fossil diesel. It is eco-friendly, non-toxic, a greener fuel and sustainable. As per our knowledge, there have been few publications in this area where the method of conversion is complicated. The aim of this study is to develop a cheap and simple method for synthesis of activated carbon from waste plastic bottles and its applicability for green fuel production from waste feedstocks (i.e., PFAD and WCO).

## 2. Materials and Methods

### 2.1. Materials

Methanol, acetone, NaOH with the purity >98 wt.%, KOH and H_2_SO_4_ were used in this study. All of these chemicals were supplied by Sigma-Aldrich (St. Louis, MO, USA). Waste PET bottles were collected from the local restaurant’s disposal areas from Serdang, Selangor, Malaysia. All the collected bottles were colorless, after the removal of the labels, detachment of the caps, washing with water to remove any dirt and drying in the oven at 70 °C, for 24 h. The dried bottles were then cut into small pieces and shredded into flakes for further processing.

### 2.2. Synthesis of Activated Carbon from Waste PET Bottles

**Figure 1:**
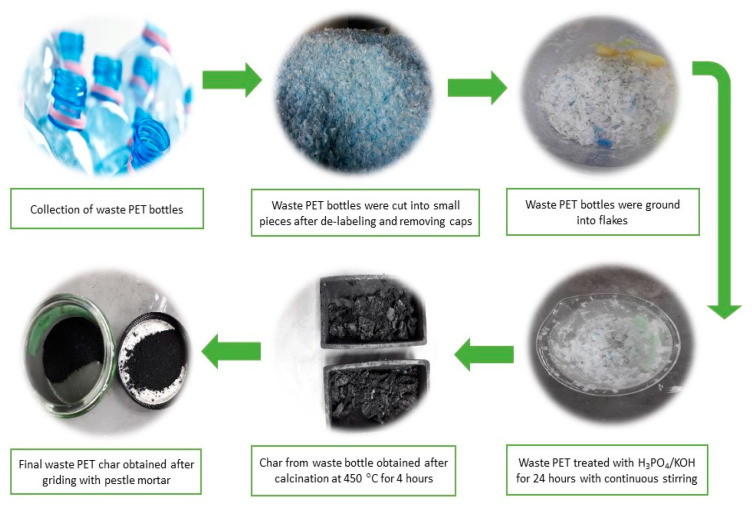
Synthesis of activated char from waste drinking bottles.

Activated carbon (AC) was prepared by carbonizing PET bottles by cutting them into small pieces of 1 cm by 1 cm dimensions and then further into small granules of 1 mm size by grinding them in a mechanical grinder for 15 min. PET granules obtained were carbonized in a stream of nitrogen, at a heating rate of 3 °C/min, temperature equal to 450 °C for 4 h to obtain AC. However, for PET H_3_PO_4_ and PET KOH, the sample was first pretreated with continuous stirring by a mechanical stirrer in H_3_PO_4(aq)_ and KOH(s) for 12 h before being subjected to pyrolysis, under the same conditions as described above. The char was obtained thoroughly washed with distilled water to remove excess acid or base and checked for the same by using a pH meter. The sample was then dried in an oven at 100 °C for 24 h (Figure 1).

### 2.3. Characterization of Activated Char

The crystal structure was determined by X-ray diffractometer (Shidmazu Corporation, Tokyo, Japan; model XRD 6000), using CuKα radiation (λ¼ 0.15406 nm, 40 kV, 30 mA). The specific surface area of sample was determined by using adsorption–desorption measurements, using Thermofinnigan Sorpmatic 1990 series. Before N_2_ sorption analysis, the samples were dried and degassed at 65 and 100 °C for 12 h, in a vacuum oven (10 Pa), using P_2_O_5_ as a water adsorbent. Based on adsorption data in the relative pressure (P/P0) range of 0.05–0.31, the specific surface area was calculated by using the multipoint Brunauer–Emmett–Teller (BET) method. The Pore Size Distribution (PSD) was assessed by using a method based on Density Functional Theory (DFT). The acidity and the basicity of the sample were studied by temperature-programmed desorption, using a Thermofinnigan TPD/R/O 1100 instrument equipped with thermal conductivity detector (TCD). The 70 mg samples were pretreated in a quartz tube reactor for 60 min, at 150 °C, with a nitrogen-gas flow (30 mL/min) to remove moisture, and then cooled to room temperature before the experiment. Following that, H_2_ gas was added to the samples at a flow rate of 25 mL/min to adsorb hydrogen, and then Ar gas was flushed through the sample (at a rate of 25 mL/min) to remove surface H_2_. At temperatures ranging from 50 to 950 °C, with a 10 °C/min ramp, the following desorption of H_2_ was observed, using a thermal conductivity detector. FTIR analysis was performed by using a Perkin–Elmer Spectrum (PS) 100 FTIR Spectrometer with a resolution of 4 cm^−1^, operating in the range of 300–4000 cm^−1^ for determination of the functional groups. The FESEM images were recorded on a Leo 1455 VP electron microscope. 

### 2.4. Application of Biodiesel Conversion

PET H_3_PO_4_ and PET KOH was used to test for the conversion of PFAD and WCO into biodiesel by following the method described [14]. The amount of conversion of PFAD or WCO into biodiesel was calculated by titration method which involves dissolving sodium hydroxide into isopropyl alcohol for titrating the solution of biodiesel which has been separated from the catalyst after centrifuging it at a speed of 3000 rpm for 20 min. The amount of FFAs present in the original PFAD and WCO was also determined by using titration. The percent conversion was performed by using the following formula:

Conversion (%) = [(AVf − AVp)/AVf] × 100%

where AVf and AVp stand for the acid value of the feedstock and acid value of the product, respectively. 

## 3. Results and Discussion

### 3.1. Field Emission Scanning Electron Microscopy (FESEM) Evaluation

**Figure 2:**
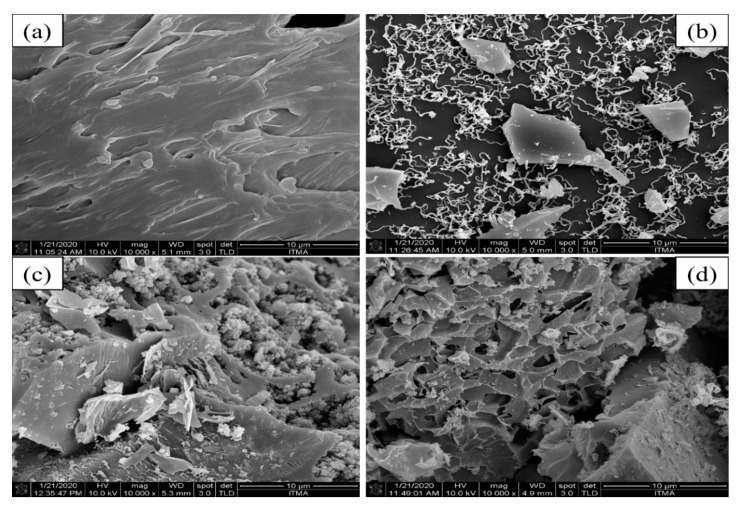
Morphological FESEM images of Raw PET and activated carbon from PET (10,000× magnification): (**a**) PET Raw, (**b**) PET Plain, (**c**) PET H_3_PO_4_ and (**d**) PET KOH.

FESEM analysis was used to study the morphological structure and elemental composition of the PET Raw, PET Plain, PET H_3_PO_4_ and PET KOH, as shown in Figure 2a–d. Figure 2a shows that external surface morphology of PET Raw, which appears as sheets with rough surface. The same behavior of rough surface was observed by Akinfalabi et al. [13]. Interestingly, PET Plain surface transformed after the calcination process and exhibited a nanotube structure with large irregularly shaped particles (Figure 2b), which is ascribed to the effect of sintering during high calcination temperature (450 °C). In contrast, Figure 2c,d highlights the effect of the treatment process of PET Plain with H_3_PO_4_ and KOH. Obviously, the chemical treatment led to the creation of pores, which are more pronounced in the case of KOH (Figure 2c). On the other hand, PET H_3_PO_4_ had an agglomerated surface, which relates to the attachment of phosphonyl groups on the catalyst surface, giving it acidity. Malinas et al. [15] prepared the activated carbon and observed the rough surface and textural nature of the activated carbon.

### 3.2. N_2_ Adsorption and Desorption Evaluation

**Figure 3:**
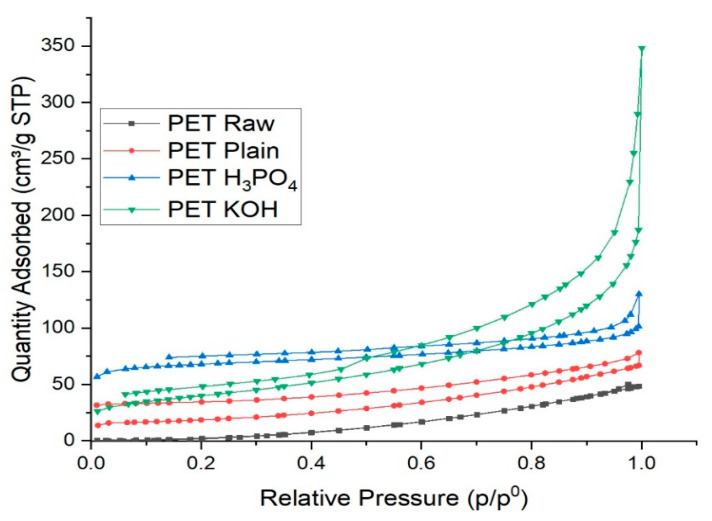
N_2_-adsorption and desorption isotherms of PET Raw, PET Plain, PET H_3_PO_4_ and PET KOH.

The adsorption isotherms of PET Raw have few values at the region of small relative pressure. Therefore, the sample can be presumed to have few micropores. The adsorption isotherms of after the heat treatments, such as PET Plain, PET H_3_PO_4_ and PET KOH, indicated the gap at the right end of the graph (Figure 3), that is, near 1 of relative pressure. 

The reason for the gap might be the absorption of the probe gas species. The adsorbed volume at small relative pressure of PET H_3_PO_4_ was larger than that of PET KOH. Therefore, the H_3_PO_4_ activation process should introduce more micropores than the KOH activation process. In the adsorption isotherm of PET KOH, the existence of mesopores could be presumed from the discontinuity at ca. 0.5 of relative pressure. 

The surface area, pore volume and diameter are presented in Table 1; the raw material and synthesis materials were determined by using BET. The surface area of the raw PET was 24 m^2^/g, which increased when the PET converted into AC after calcination to 65 m^2^/g. This shows that the calcination changes the nature of the raw PET, and when PET is exposed to a higher temperature, its surface changes and creates more pores, and this behavior confirms the previous studies [9]. Interestingly, the treated carbon PET H_3_PO_4_ and PET KOH revealed a high surface area compared to raw material 140 and 261 m^2^/g, respectively. In the case of pore volume, the PET Raw displayed a minimum pore volume value of 0.06 cc/g. Conversely, the pore volume of PET Raw and treated PET H_3_PO_4_ and PET KOH dramatically increased due to the formation of oxides by KOH during pyrolysis. The pore radius of PET Raw was 5.02 nm, wherein it was marginally enlarged to 5.92 nm in PET KOH, ascribed to the collapse of pore walls in the calcination and the chemical-treatment process. It was discovered that activating phosphoric acid might stimulate the creation of pores and enhance the specific surface area [16]. On the contrary, the pore radius PET H_3_PO_4_ decreased as a result of the blocking of pores by phosphonyl groups of H_3_PO_4_. The pore diameter in all the samples was 18 nm, demonstrating the presence of mesopores. The BJH model was used to calculate mesopore volume at 0.1–1.0 p/p0, and it was found that all the chars had well-developed mesopore volume. The mesopore volume distribution confirmed in previous studies [17]. It can be seen from Table 1 that the pore formation is better in PET KOH as compared to others and basicity of the PET KOH is higher than the other impregnated AC. 

### 3.3. Temperature-Programmed Desorption of Carbon Dioxide and Ammonia (TPD-CO_2_/NH_3_)

The acidity and basicity of the synthesized activated carbon catalysts were analyzed by using temperature programmed desorption, using ammonia and carbon dioxide as probe gases (TPD-NH_3_ and TPD-CO_2_), respectively. PET H_3_PO_4_ was found to have higher acidity, i.e., 18.17 mmol g^−1^, due to the attachment of phosponyl groups to it during pretreatment, whereas, in case of PET KOH, the basicity increases to 13.49 mmol g^−1^ (Table 1), due to the formation of oxides by KOH during pyrolysis. H_3_PO_4_ plays a dual role in the introduction of phosponyl groups into the structure and creation of pores, whereas KOH creates pores in the structure and imparts basicity to it by the formation of oxides of potassium. Similar results were reported in the previous studies [18]. 

### 3.4. Material’s Thermal Stability Analysis Using Thermogravimetric Analysis (TGA)

**Figure 4:**
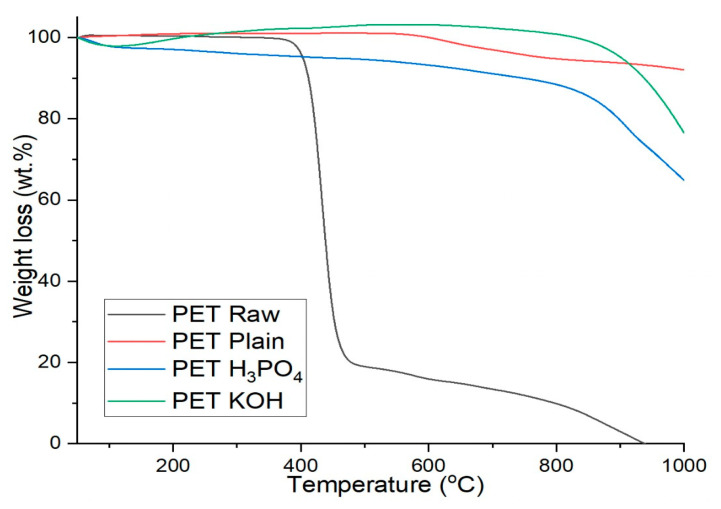
TGA curve of PET Raw, PET Plain, PET H_3_PO_4_ and PET KOH.

In this work, thermogravimetric analysis was carried out to analyze the thermal stability and decomposition rate of PET Raw, PET Plain, PET H_3_PO_4_ and PET KOH. Figure 4 shows that the gradual weight loss of PET at different temperatures. A significant weight loss started at 360–490 °C, which is due to the degradation of the PET components (first degradation). The second degradation stage occurred at 500 °C with very slow change and completed at around 950 °C. The TGA of PET Plain shows that there is an initial increase in weight due to the absorption of moisture. Meanwhile, weight loss is seen at a temperature of 530 °C, which indicates that the sample is quite stable, and it continues to decompose till 950 °C, with a total weight loss of 92% of PET polymer, which is associated with the thermal decomposition of the carbon sheet’s structure [19]. On the other hand, the TGA of PET H_3_PO_4_ exhibited marginal weight reduction attributed to the moisture loss at around 70 °C. At 90 °C, the sample starts to show an increase in weight loss that continues till 600 °C, being due to the breakdown of phosphoryl group. Notably, at 830 °C, the PET H_3_PO_4_ starts further decomposition, and the weight loss is due to the breakdown of activated carbon. Remarkably, the TGA of the PET KOH shows an excellent stable characteristic from 50 to 800 °C. Beyond 800 °C, the catalyst starts to decompose with the mass loss being around 10%, which indicates that KOH play a vital role in stabilizing and decomposition rate of the PET KOH. 

### 3.5. Crystallinity Evaluation via XRD Analysis

**Figure 5:**
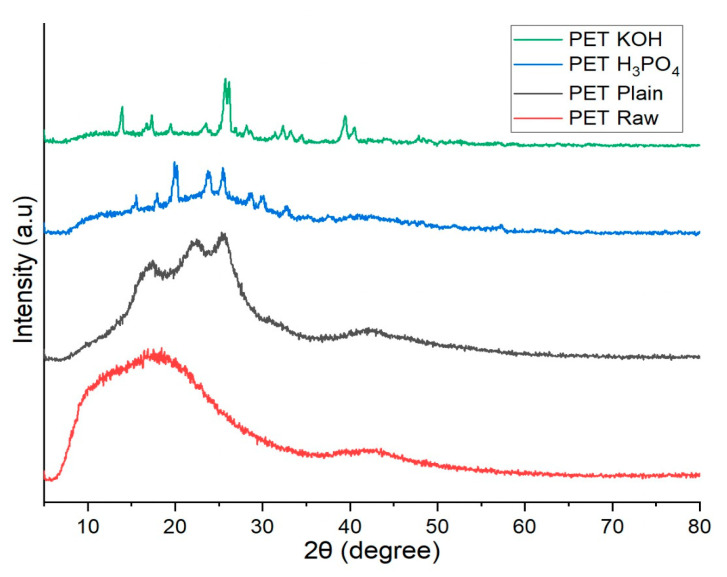
X-ray diffraction (XRD) spectra of PET Raw, PET Plain, PET H_3_PO_4_ and PET KOH.

The XRD spectra of PET Raw, PET Plain, PET H_3_PO_4_ and PET KOH are presented in Figure 5. Amorphous carbon peaks were obvious in the spectra from the PET Raw, PET Plain and PET H_3_PO_4_. After pretreatment with KOH and subsequent carbonization, K and K_2_O were observed in the char, giving an indication that KOH had reacted with the carbonaceous components in PET. Only amorphous carbon was detected in PET H_3_PO_4_, indicating that the changes due to H_3_PO_4_ mainly affected the organic components of PET [20]. 

### 3.6. Functional Groups Evaluation via FTIR

**Figure 6:**
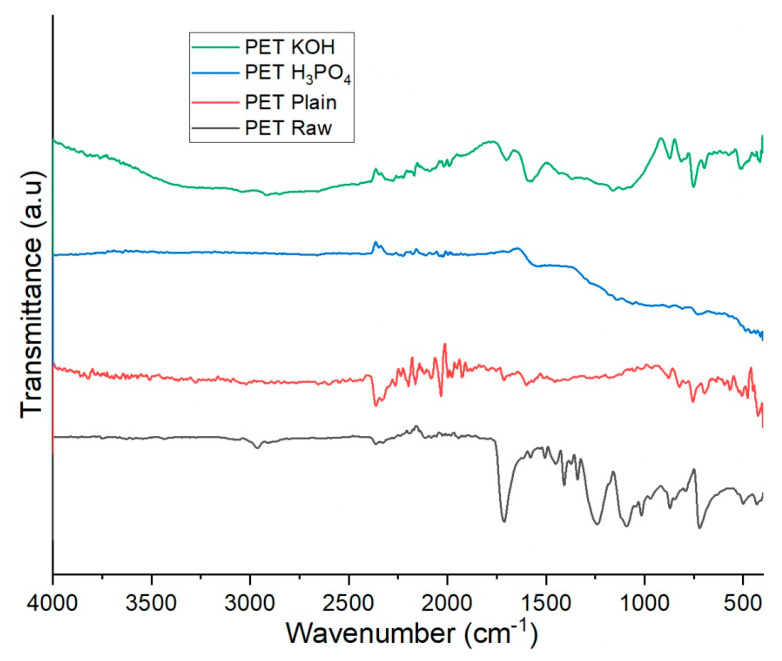
FTIR spectra of PET Raw, PET Plain, PET H_3_PO_4_ and PET KOH.

The existence of functional groups in PET Raw, PET Plain, PET H_3_PO_4_ and PET KOH was determined by using FTIR (Figure 6). The observed spectra are fundamental to carbon materials. The infrared spectrum of PET Raw (-OCH_2_CH_2_OOCC_6_.H_4_CO-), has been studied from longtime. For the PET Raw, five main peaks are identified at wavenumbers 1715, 1245, 1100, 870 and 730 cm^−1^, corresponding in ketones (C=O), ether aromatic (C–O), ether aliphatic (C–O), aromatic (C–H) and aromatic (C–H) bond [21]. FTIR spectra were obtained for the PET Raw sample and PET KOH. In the range of 3400–3500 cm^−1^, a broad and powerful peak is found, implying stretching vibrations of –OH (hydroxyl) groups and adsorbed moisture. In activated samples, this band moves to a higher wavenumber (3445–3456 cm^−1^) due to the formation of hydrogen bonds after KOH pretreatment [19]. The stretching of methylene (–CH_2_) and methyl (–CH_3_) results in a tiny absorption band around 2900 cm^−1^. Peaks about 1600 cm^−1^ are connected to the carboxylate and carbonyl groups. The existence of oxygen moieties is confirmed by a prominent peak at 1013 cm^−1^ for PET Raw and 3–700, which is connected to C–O–C stretching in aromatic compounds. The peak at 2300 cm^−1^ corresponds to C–H sp3 stretching, while the existence of peaks in the 690–762 cm^−1^ range is a benzene ring characteristic [22]. The absorption peaks due to stretching vibration of C–H on the benzene ring and C=O on PET H_3_PO_4_ were at 725 and 1705 cm^−1^, respectively. The weak absorption peak at 969 cm^−1^ of the PET H_3_PO_4_ spectrum was attributed to P–O–C groups, which were formed by the reaction between polyester and phosphoric acid [20].

### 3.7. Application of PET H_3_PO_4_ and PET KOH for Green Fuel Production

The prepared PET H_3_PO_4_ and PET KOH were employed for conversion of PFAD and WCO, respectively, into esters. The PFAD gives 45% conversion when using PET H_3_PO_4_, whereas WCO gives 50% conversion yield when the reaction is performed by using the PET KOH, as shown in Table 1. These results shows that both PET H_3_PO_4_ and PET KOH have the potential to be used for the production of green fuel. By using these materials, the overall production cost can be reduced, and eventually, the final price of ester could be cheaper. There is still a need to optimize the green-fuel production process by using these materials. 

## 4. Conclusions

In this study, activated carbon derived from discarded plastic drinking bottles was converted into acidic and basic char and tested for the feasibility of conversion of the PFAD and WCO into esters. The porous structure and increase in acidity or basicity developed on the surface of PET Plain were produced after pretreatment of H_3_PO_4_ and KOH. The maximum surface area was found in the case of PET H_3_PO_4_, having an acidity of 18.17 mmol g^−1^. That one gives a conversion of 45%, whereas, in the case of PET KOH, the basicity of 13.49 mmol g^−1^ gives a conversion of 50%. Further improvement in the yield can be obtained by treating the PET Plain with various agents, such as metal oxides; metal nitrates; and sulfonating agents, e.g., sulphuric acid and chlorosulfonic acid, to convert it into an efficient catalyst. The PET-based char can be used for different purposes, such as carbon-based precursors, in adsorbents preparation and in the treatment of laundry water for removal of microplastics from it. Waste PET char has an interesting scope to solve the problem of environmental pollution due to non-biodegradable wastes which are dumped normally and change the focus from food and energy controversy.

## Figures and Tables

**Table 1:** Textural properties and total desorption of CO_2_ and NH_3_ for PET Raw, PET Plain, PET H_3_PO_4_ and PET KOH.

Materials	S*_BET_* (m^2^ g^−1^) *	D*_p_* (nm) **	V*_p_* (cm^3^ g^−1^) ***	TPD-CO_2_	TPD-NH_3_	Conversion (%)
				Total Basicity (mmol g^−1^)	Total Acidity (mmol g^−1^)	
PET Raw	24	5.02	0.06	ND	ND	ND
PET Plain	66	5.32	0.10	1.69	3.99	ND
PET H_3_PO_4_	261	4.71	0.15	0.17	18.17	45
PET KOH	141	5.92	0.24	13.49	3.50	50

* BET specific surface area; ** average pore diameter; *** total pore volume at p/p0 = 0.99; ND = not determined.

## Data Availability

Not applicable.
